# Comparison of SCAphoid fracture osteosynthesis by MAGnesium-based headless Herbert screws with titanium Herbert screws: protocol for the randomized controlled SCAMAG clinical trial

**DOI:** 10.1186/s12891-019-2723-9

**Published:** 2019-08-07

**Authors:** Sören Könneker, Katja Krockenberger, Claudia Pieh, Christian von Falck, Bernard Brandewiede, Peter M. Vogt, Martin H. Kirschner, Andreas Ziegler

**Affiliations:** 1 Department of Plastic, Aesthetic, Hand and Reconstructive Surgery, Hanover Medical School (MHH), Carl-Neuberg-Straße 1, 30625 Hannover, Germany; 2 Amedon GmbH, Willy-Brandt-Allee 31c, 23554 Lübeck, Germany; 3 Syntellix AG, Aegidientorplatz 2a, 30159 Hannover, Germany; 4 Institute for Diagnostic and Interventional Radiology, Hanover Medical School, Carl-Neuberg-Str. 1, 30625 Hannover, Germany; 5 Department for General, Trauma and Reconstructive Surgery, Ludwig-Maximilians University of Munich, Marchioninistraße 15, 81377 Munich, Germany; 6 StatSol, Moenring 2, 23560 Lübeck, Germany; 7 School of Mathematics, Statistics and Computer Science, University of KwaZulu-Natal, Pietermaritzburg, South Africa

**Keywords:** Herbert screw, Magnesium alloy, Magnetic resonance imaging, Non-inferiority, Patient-rated wrist evaluation, Quality of life, Scaphoid fracture, Titanium alloy

## Abstract

**Background:**

Scaphoid fractures are the most common carpal fractures. They often need to be treated by surgery, where the use of a compression screw is the globally accepted gold standard. Surgeons may choose between different implant materials including titanium alloys, which remain in the body or are removed after healing. An alternative are biodegradable magnesium-based implants. Properties of magnesium alloys include high stability, osteoconductivity, potential reduction of infections and few artifacts in magnetic resonance imaging (MRI). The aim of this trial is to demonstrate non-inferiority of magnesium-based compression screws compared with titanium Herbert screws for scaphoid fractures.

**Methods:**

The trial is designed as a multicenter, blinded observer, randomized controlled parallel two-group post market trial. Approximately 190 patients will be randomized (1:1) with stratification by center either to titanium or magnesium-based compression screws. Follow-up is 1 year per patient. Surgical procedures and aftercare will be performed according to the German treatment guideline for scaphoid fractures. The first primary endpoint is the patient-rated wrist evaluation (PRWE) score after 6 months. The second primary endpoint is a composite safety endpoint including bone union until 6 months, no adverse device effect (ADE) during surgery or wound healing and no serious ADE or reoperation within 1 year. The third primary endpoint is the difference in change MRI artifacts over time. Non-inferiority will be investigated for primary endpoints 1 (t-test confidence interval) and 2 (Wilson’s score interval) using both the full analysis set (FAS) and the per protocol population at the one-sided 2.5% test-level. Superiority of magnesium over titanium screws will be established using the FAS at the two-sided 5% test-level (Welch test) only if non-inferiority has been established for both primary endpoints. Secondary endpoints include quality of life.

**Discussion:**

This study will inform care providers whether biodegradable magnesium-based implants are non-inferior to standard titanium Herbert screws for the treatment of scaphoid fractures in terms of wrist function and safety. Furthermore, superiority of magnesium-based implants may be demonstrated using MRI, which is used as surrogate endpoint for screw degradation.

**Trial registration:**

DRKS, DRKS00013368. Registered Dec 04, 2017.

**Electronic supplementary material:**

The online version of this article (10.1186/s12891-019-2723-9) contains supplementary material, which is available to authorized users.

## Background

The fracture of the scaphoid is the most common carpal fracture accounting for 2 to 7% of all fractures [1], and young men between 20 and 29 years are predominantly affected [2]. This has an economic impact on the society caused by the absence from work. Therefore, the goals of treatment are to avoid long immobilization, bone healing disorders i.e., pseudarthrosis of the scaphoid and to gain normal wrist function.

The probability for bone healing mainly depends on displacement and location of the fracture and decreases substantially if the fracture gap width exceeds 2 mm [3]. Surgical treatment is generally recommended for unstable fractures, which include fractures with displacement ≥1 mm or ≥ 2 mm, comminuted fractures, perilunate fracture-dislocation and all fractures in the proximal third because of a long immobilization time and a high risk for pseudarthrosis [4]. Conservative treatment is often recommended for stable fractures [5]. Specifically, the German treatment guideline for scaphoid fractures [6] recommends conservative treatment of tubercle fractures (type A1) and non-dislocated transverse fractures in the middle or distal third (type A2) for the classification scheme by Herbert and modified by Krimmer et al. [4]. Recommendations for operative treatment have, however, been expanded in recent years to include non-displaced waist fractures to avoid long periods of immobilization and by that resulting joint stiffness [5], and the German treatment guideline recommends minimal invasive surgery for A2 fractures.

Headless compression screws are considered to be particularly well suited for surgical therapy of recent fractures [6], which can be inserted through both palmar and dorsal approaches. Most frequently, non-biodegradable metallic implants are used, specifically titanium or steel alloys. However, surgeons may also choose absorbable implants, e.g., polymer-based implants [7] and magnesium-based implants [8, 9], which have the clear advantage that they degrade over time so that no second surgery is required for implant removal [10, 11]. Screw removal rates between 8 and 14% have been reported for various indications in the literature [12–14]. A major advantage of magnesium-based implants over polymer implants is their higher stability [8, 9]. They also have osteoconductive properties [9] and are supposed to inhibit infections [9].

### Few artifacts in magnetic resonance imaging and computed tomography scans

Furthermore, magnesium-based implants produce almost no artifacts in MRI or CT even when imaging was done immediately after implantation [9, 15]. In contrast, titanium screws generally create interference. On the 3 T scanner employed by Sonnow et al. [15], the largest difference in artifacts between magnesium-based and titanium implants was observed when imaging was done with the T1w turbo spin echo (TSE) sequences. Fewer artifacts were also observed for the proton density weighted (PDw) TSE and PDw TSE metal-artifact reduction (WARP) sequences. However, differences were less pronounced.

Magnesium-based implants thus better facilitate post-operative follow-up. In addition, since magnesium-based implants are biodegradable, a further reduction of artifacts over time is expected. Change of artifact size over time may thus serve as surrogate marker for screw degradation.

### Magnesium-based compression screw

**Fig. 1:**
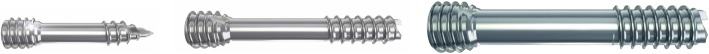
Magnesium-based compression screws to be used in this trial. From left to right: MAGNEZIX® CS 2.0, MAGNEZIX® CS 2.7, MAGNEZIX® CS 3.2

MAGNEZIX® CS of Syntellix AG are magnesium-based Herbert screws. The alloy is based on the MgYREZr/WE43 system and contains more than 90% magnesium and, in addition, yttrium, rare earth metal and zirconium. It is free of aluminium, a well-known neurotoxin [16]. MAGNEZIX® CS has market approval for the European Union, countries belonging to the European Free Trade Association and 21 additional countries worldwide (as of July 31, 2018). The small CS 2.0 is not cannulated, while CS 2.7 and CS 3.2 are cannulated to allow insertion of a guide wire (Fig. 1). All screw models have a self-cutting tip to ease insertion. During screw implantation, specially modified instruments are used.

### Radiolucent zone around any part of the implant

Radiolucent zones around magnesium-based implants have been described in the literature [17, 18]. Although this may be visually inconvenient, this effect is only short-term, it does not affect bone healing and disappears automatically over time. Experiences from laboratory testing animal studies have shown the disappearance of the screw within 1 year [17, 18], and in a clinical trial it was degraded after 3 years [19].

In summary, radiolucent zones are expected during the degradation of the screw because of the material properties of magnesium and its degradation via corrosion. Consequently, this effect is not taken account as a screw-related complication.

### Breakage of magnesium-based screws in the healing process

When the healing process is well advanced, MAGNEZIX® implants will be recognized as being deformed in diagnostic imaging caused by the degeneration process of the implant. In this process, these implants did not break because of a lack of stability, but they were degrading as intended, while the bone continued to heal and gradually bore higher load capacity.

In summary, the breakage of MAGNEZIX® CS during the healing process may occur due to the degradation process. Consequently, this effect is not considered a screw-related complication.

### Clinical evidence of magnesium-based screws

In an RCT, biodegradable magnesium-based screws were not inferior to titanium screws for the treatment of mild hallux valgus deformities [20]. Primary endpoint of this trial was the absolute difference between the distal metatarsal articular angle (DMAA) measured 6 months post-surgery and immediately post-surgery. The authors also measured the American Orthopedic Foot and Ankle Society (AOFAS) score for hallux, visual analog scale (VAS) for pain assessment and range of motion (ROM) of the first metatarsophalangeal (MTP-IP) joint. The authors observed comparable values between titanium and magnesium-based screws for these outcome variables. In addition, no foreign body reactions, osteolysis and systemic inflammatory reactions were detected. During the implants’ absorption process, no increase of the laboratory values of electrolytes including magnesium or other components or degradation products of the magnesium-based screw were found [20]. Three-year follow-up data to this trial have been reported [19].

Magnesium-based compression screws are meanwhile used in several indications, e.g., for the treatment of hallux valgus [19–22], malleolar fractures [23, 24], ankle joint fractures [25], in the treatment of fibula fractures [25], scaphoid fractures [26, 27] or radial head fractures [28].

### The need for a trial

Windhagen et al. [20] focused on elective foot surgery. However, an RCT for the use of magnesium-based screws is lacking in fractures, especially in scaphoid fractures. This trial is required to show the non-inferiority of magnesium-based screws compared to conventional titanium screws in hand and trauma surgery.

### Study objectives and hypotheses

The general aim of this trial is to obtain precise, well-founded and systematic application data of biodegradable magnesium-based compression screws compared to conventional titanium compression screws for the treatment of scaphoid fractures.

Specifically, we hypothesized that biodegradable magnesium-based compression screws are non-inferior compared with titanium screws in the treatment of isolated scaphoid fractures in terms of both efficacy and safety. In addition, we hypothesize that magnesium-based compression screws are superior to titanium screws in image analysis because magnesium-based compression screws are biodegradable.

The primary objective of this trial thus is to determine non-inferiority for efficacy and safety of magnesium-based compression screws when compared to titanium screws in the surgical treatment of patients with scaphoid fracture. If non-inferiority can be shown for both efficacy and safety, the final primary aim is to demonstrate superiority of magnesium-based compression screws over titanium screws with the surrogate variable artifact reduction as indicator for degradation of magnesium-based compression screws in-vivo.

Secondary objective of the trial is to compare quality of life in patients treated with magnesium-based compression screws with classical Herbert titanium screws.

## Methods

### Study design

SCAMAG is a randomized, controlled, parallel-group, open-label with blinded observer, multicenter trial with two groups for comparing biodegradable magnesium-based screws with standard titanium Herbert screws. The study protocol follows the SPIRIT statement [29]. Reporting will follow the CONSORT statement [30] and its extension to abstracts [31].

Following the SPIRIT statement, we have created Additional file 1 to show the proposed participant flow through the study. The SPIRIT checklist is shown in Additional file 2.

The study was approved by the ethics committee of the Hanover Medical School (MHH) on September 27, 2017 (registration number: 7614), and it was registered with the German Register for Clinical Trials (DRKS, drks.de) on Dec 04, 2017 (registration number: DRKS00013368).

### Setting, recruitment, inclusion and exclusion criteria

After diagnosis of a scaphoid fracture and when a decision for the indication of an osteosynthesis with compression screw is made, patients will be recruited by the surgeon in 13 high-volume centers in Germany. Each center has experience in conducting clinical trials, treating patients with scaphoid fractures and the use of both MAGNEZIX® CS compression screws and titanium Herbert screws. All surgeons are trained in fixation of scaphoid fractures with titanium Herbert screws and MAGNEZIX® CS compression screws prior to the trial.

Study centers will receive a case payment of 500 € in two parts. The first payment in the amount of 50% will be paid after surgery and the second part after 12 months, if complete follow-up data is available.

Inclusion and exclusion criteria are displayed in Table 1.

**Table 1:** Inclusion and exclusion criteria

Inclusion criteria: • Indication for screw fixation of scaphoid fracture which is not older than 12 weeks; classification by Herbert, modified by Krimmer of types A2, B1, B2, B3 [4], • normal wrist function prior to fracture, • age ≥ 18 years, • written informed consent for trial participation and surgery. Exclusion criteria: • Previous surgery on the wrist, associated injuries, state after or suspicion of complex regional pain syndrome (CRPS), • simultaneous fractures of the forearm of both sides and those who will influence the postoperative care, • known ligamentary concomitant injuries of the wrist on both sides and those who will influence the post-operative care, • radiological findings of medium to high grade osteoporosis, • intended or conducted spongiosa transplantation or bone graft transplantation during surgery, • pregnancy, suspected pregnancy or breastfeeding period, • allergies to components of osteosynthesis material, • participation in other clinical trials up to 30 days before inclusion in this trial, • central neurological deficits which do not permit a compliance to the trial, especially during follow-up, • for patients recruited to the centers near Hanover and undergoing MRI: claustrophobia and metallic implants which are contraindicative for an MRI.	

### Intervention: magnesium-based screws

Usual preoperative preparation will be performed. The biodegradable compression screw MAGNEZIX® CS will be used in the following dimensions: MAGNEZIX® CS Ø 2.0 mm, length 8 mm to 24 mm in 2 mm stages, cannulated MAGNEZIX® CS Ø 2.7 mm, length 10 mm to 34 mm in 2 mm stages and cannulated MAGNEZIX® CS Ø 3.2 mm, length 10 mm to 40 mm in 2 mm stages (Fig. 1).

Surgical procedures will be done as established in the study center with minimal access as possible: percutaneous, minimal invasive or open approach depending on the fracture type. After surgery the success of reposition and osteosynthesis will be controlled via plain radiographs. The further treatment and aftercare will be performed strictly as recommended in the AWMF S3 guideline for scaphoid fractures according to the fracture type: no immobilization for stable fractures, 4 weeks of immobilization for waist fractures and 6 weeks of immobilization for proximal pole fractures [6].

According to the fracture type splinting and training will be performed with x-ray control. In case of unclear fracture healing as assessed by x-rays, a CT-scan will be done to determine fracture healing but, according to the AWMF S3 guideline for scaphoid fractures [6] at the earliest 9 weeks after surgery.

### Control: titanium Herbert screws

Usual preoperative preparation will be performed. Classical titanium Herbert screws will be used in the control group. Size of screws and manufacturer are chosen by the surgeon.

All other procedures are identical as in the magnesium group.

### Baseline and follow-up examinations

**Fig. 2:**
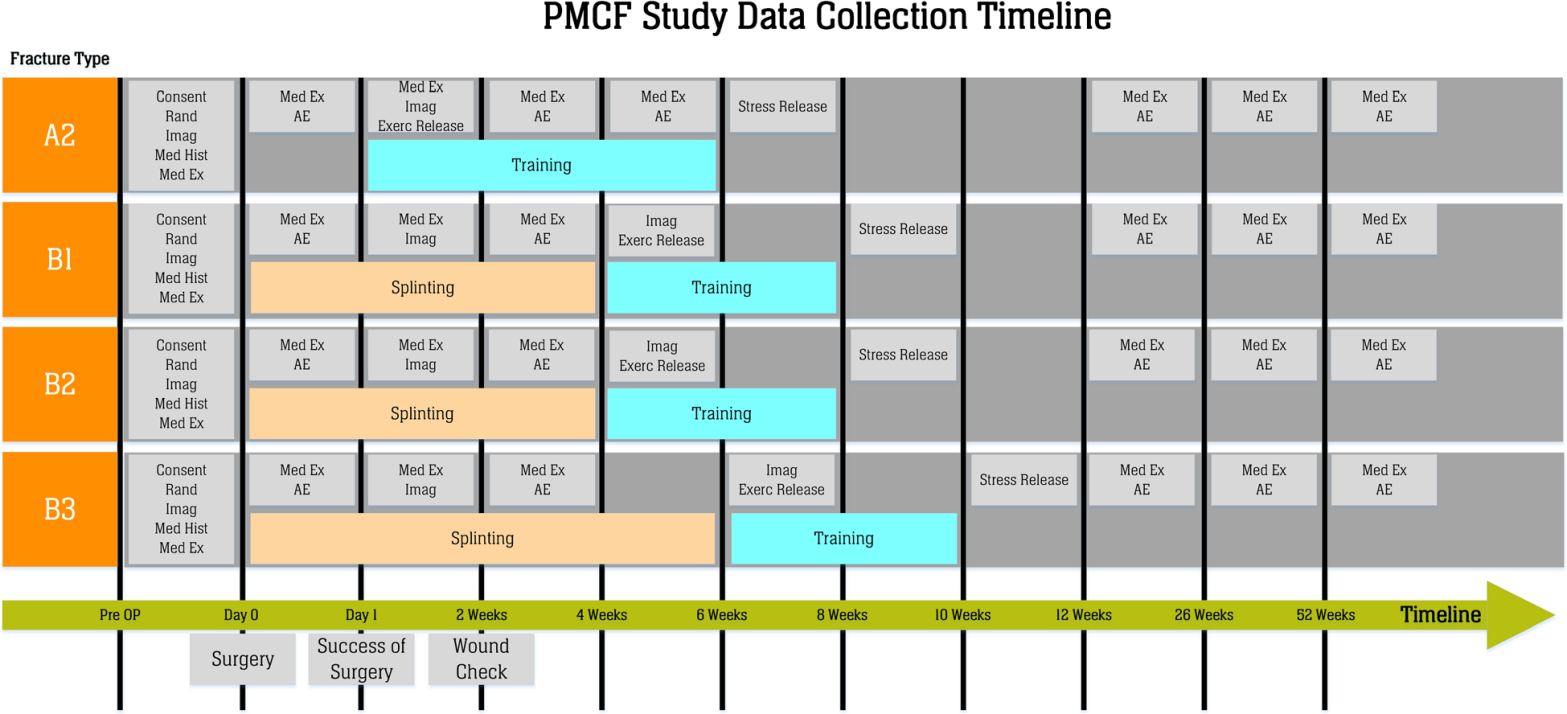
Trial flow by fracture type. Patients in group A2 start with exercises immediately after surgery, and there is not specific exercise release. Abbreviations used in figure: Consent: informed consent; Rand: randomization; Imag: imaging, i.e., X-Ray and CT-scans according to guideline; Med History: medical history; Med Ex: medical examination; AE: adverse event; Exerc Release: exercise release

An overview of scheduled study visits is shown in Table 2. A baseline examination will be prior to surgery after having obtained informed consent. Success of surgery will be checked, in general, at day 1. Wound check is scheduled 2 weeks after surgery. Patients with scaphoid fractures grades B1 to B3 will be seen prior to exercise release. All patients will be seen prior to stress release. Time for exercise and stress release depend on the fracture type (Fig. 2). Before exercise release and before stress release after the period of exercising, clinical examinations will be performed.

**Table 2:** Schedule of assessments

	Baseline	Surgery	Success of surgery at day 1	Wound check	Exercise release	Stress release	Follow-up at		
							3 months	6 months	12 months
In- and exclusion criteria	X								
Informed consent	X								
Randomization	X								
Medical history	X								
Medical examination	X	X	X	X	X	X	X	X	X
Adverse events		X	X	X	X	X	X	X	X
EQ-5D-5 L	X						X	X	X
PRWE							X	X	X
DASH							X	X	X
KWS							X	X	X
X-ray / CT (according to guideline)	X		X		X	X	(X)		
Range of motion						X	X	X	X
Grip strength						X	X	X	X
Pain NAS			X	X	X	X	X	X	X

*EQ-5D-5 L* Quality of life; *PRWE* patient-rated wrist evaluation, *DASH* Disabilities of the Arm, Shoulder and Hand, *KWS* Krimmer Wrist Score, *NAS* Numeric analogue scale

Patients will be followed up at 3 months, 6 months and 12 months after randomization. Data will be collected by a physician and a nurse who will both be blind to the randomization status. The follow-up period will 12 months after randomization.

### Magnetic resonance imaging (MRI) group of the trial

Twenty randomized patients from the centers located in or near Hanover – 10 patients with titanium screws and 10 patients with MAGNEZIX® CS – will additionally receive MRI imaging 6 weeks, 3, 6 and 12 months after randomization. MRI scans will be performed on a 3 T scanner located within the MHH.

The scanning protocol will include the following sequences: proton density weighted (PDw) turbo spin echo (TSE) fat saturated (FS) sequences in coronal and axial planes (slice thickness ≤ 2 mm), T2w TSE coronal (1 mm, without FS) and sagittal (≤2 mm, FS), T1w TSE coronal (≤2 mm, without FS) and a T1w TSE oblique sagittal sequence (1 mm, without FS) adjusted to the scaphoid bone. There will be no gap between the slices and the field of view (FOV) will not exceed 10 × 10 cm. No intravenous contrast agent will be used. All images will be viewed in a preset window-level and width with the following values for window center and width (C/W): PDw TSE/T1w 650/1300; PDw WARP 400/800.

### Primary endpoints

Three primary endpoints will be used in this trial. The first primary outcome will be the German version (PRWE-G) of the patient-rated wrist evaluation (PRWE) total score measured 6 months after randomization [32]. The PRWE is a 15-item questionnaire which is completed by the patient. It is a brief, reliable and valid instrument for assessing wrist pain and disability [33, 34]. Scoring for all the questions is on a 10-point ordered scale ranging from ‘no pain’ or ‘no difficulty’ (0) to ‘worst ever pain’ or ‘unable to do’ (10). Two non-overlapping domains pain (5 items) and function (10 items) are generated. Pain and function scores are converted to scales of 0–50 and are summed for a total score, which means that both domains are weighted equally. In case of a missing item, it has been recommended to replace the item with the mean score of the domain.

The PRWE has excellent test-retest reliability, validity and responsiveness [35]. Construct validity was significantly higher in PRWE compared to the short form 36 (SF-36) in patients with distal radius fracture, and it was similar to the DASH [36]. PRWE also outperformed the SF-36 in terms of responsiveness [37]. Overall, Dacombe et al. [35] concluded that the PRWE has by far the best demonstrated reliability and validity in a wrist trauma population among of all the patient rated outcome measures for the assessment of hand and wrist trauma. The PRWE has also been recommended as PRO by others, see, e.g., [38] They also concluded that the PRWE is the most responsive measure for a distal radius fracture population. It is used as primary outcome in at least two ongoing trials (ISRCTN67901257; NCT02154620).

Safety is the second primary outcome. It will be a composite endpoint, consisting of evidence of bone union until 6 months, no adverse device effect (ADE) during surgery, no ADE during wound healing, no reoperation and no serious adverse device effect (SADE) within 1 year after randomization. The outcome “safety event” thus is dichotomous and scored as 1 for a patient if

- an ADE occurred during surgery and/or
- an ADE occurred during the wound healing period and/or
- an SADE occurred during the 1-year follow-up period and/or
- a reoperation was performed during the 1-year follow-up period and/or
- there was no or incomplete bone union until 6 months after randomization, and 0, otherwise.

AEs during surgery include the following:

- Nerve events of the superficial division of the radial nerve or the median nerve,
- vessel events with large hematoma with diameter > 2 cm,
- screw related complications (fracture or bending of the screw),
- technical problems (protrusion into the adjacent joint and/or bone without correction, wrong choice of screw length) and
- revision of operation technique.

Artifacts will be measured as described by Sonnow et al. [15] to evaluate changes of artifact appearance over time. In detail, all artifacts will be assessed in an axial plane or reconstruction of the screw. In MRI artifacts aligning the y-axis will be considered, defined as the vertical axis of the scanner. As artifact appearance is expected to be symmetrical, measurement will be performed by creating a straight line through the outer boundaries of the artifacts and the central screw axis. The degree of artifact is defined as the diameter of the signal loss induced by the screw in MRI. When artifacts with various lengths are produced, the longest will be measured. This process will be performed in a total of three different axial slices of the screw, and the average value will be obtained. For better orientation and comparability, the artificial cartilage lesion will serve as reference, thus the slices in a similar position will be chosen.

The change in artifacts can now be defined as follows: Difference between maximum length of an artifact between 1-year follow-up and baseline. The third primary endpoint thus is defined quantitatively as the extent of change in artifacts. The specific imaging modality for the third primary endpoint will be defined as early as possible during the trial and before inclusion of the last patient of the MRI part of the trial.

### Secondary endpoints

Secondary endpoints include the domains pain and function of the PRWE, measured at 3, 6 and 12 months. We also use the disabilities of the arm, shoulder and hand (DASH) questionnaire [39] – total score, module scores sport/music, module score work – and the Krimmer wrist score (KWS) with its four domains pain, active flexion/extension arc, grip strength and ability to return to regular employment or activities [40], both measured at 3 months, 6 months and 12 months as secondary endpoints. Range of motion will be assessed with a commercially available goniometer (wrist flexion, wrist extension, wrist radial deviation, wrist ulnar deviation, forearm supination, forearm pronation) at months 3, 6 and 12. At the same follow-up time points, grip strength will be determined with a dynamometer.

Evidence of bone union until 6 months will be determined using x-ray images in 2 planes: anterior-posterior and lateral, in case of uncertainty additionally in a third plane, namely Stecher. Union is defined as complete disappearance of the fracture line on radiographs. If bone healing cannot be assumed in plain radiographs, CT-Scan will be performed according to the AWMF guideline for scaphoid fractures [6]. Images 6 months after surgery will be assessed independently by two experienced radiologists. Edema in MRI will be recorded at 3, 6 and 12 months. Time until return to work and recreational activities will be established through patient self-report.

Finally, quality of life will be measured with the EQ-5D-5 L [41, 42] at baseline, 3, 6 and 12 months after surgery.

### Blinding

Blinded assessment will be done for KWS, range of motion (ROM) and grip strength. Blinding will not be possible for images. The PRWE is patient reported and therefore not assessable in a blinded fashion. In addition, blinding is not possible for the assessment of the effects of the devices for the safety endpoints.

### Randomization

Patients will be randomized individually to one of the two treatment groups in a 1:1 ratio in the order they qualify. After inclusion and exclusion criteria have been checked, the presence of the informed consent form has been ticked and the form has been electronically signed by the surgeon, the randomization result will be displayed in the trial database after anesthesia. Permuted block randomization is used with variable block length stratified by center. Randomization lists for the PBR will be generated using the randomization software RITA [43]. Concealment of allocation will be guaranteed through central randomization within the electronic case report form (eCRF) according to Standard Operating Procedures (SOP).

An expert reviewer pointed out that the trial might have benefited from stratification by fracture type. The decision was to stratify by center because strata would have become very small in case of stratification by center and fracture type. Preference was given to center because of its known effect and its mentioning in the International Conference on Harmonisation (ICH) E9 guideline [44].

### Statistical analysis

All statistical analyses will be described in detail in a statistical analysis plan (SAP) which will be finalized before the randomization of the last patient. Analysis populations for the primary endpoints will be the full analysis set (FAS) based on the intention to treat (ITT) principle and the per protocol population (PP). Neither interim analyses nor adaptations are planned for this trial. All statistical analyses will be done using the R software.

The familywise error rate is set to 5%. The three primary hypotheses will be investigated hierarchically.

The hypothesis for the first primary endpoint is that the mean PRWE in the magnesium-based group is non-inferior to the mean PRWE in the titanium group, measured 6 months after randomization. The non-inferiority margin is 10 points; low values of the PRWE denote ‘no pain’ or ‘no difficulty’. The one-sided 97.5% confidence interval from the t-test will be estimated for judging non-inferiority. Sensitivity analyses will be performed using linear mixed models with center as random effect.

The hypothesis for the second primary endpoint is that the rate of safety events in the magnesium-based group is non-inferior to that in the titanium group within 1 year after surgery. The non-inferiority margin is 15%. The one-sided 97.5% Wilson score interval for the difference of two proportions will be estimated for judging non-inferiority [45]. Sensitivity analyses will be performed using logistic regression mixed models with center as random effect.

The type I error level is set to 2.5% one-sided for the first two primary endpoints. Non-inferiority will only be claimed if

1. the hypothesis for the first primary endpoint shows non-inferiority in the FAS based on the ITT population,
2. the hypothesis for the second primary endpoint shows non-inferiority in the FAS based on the ITT population,
3. the hypothesis for the first primary endpoint shows non-inferiority in the PP population and
4. the hypothesis for the second primary endpoint shows non-inferiority in the PP population.

No adjustments will be made for multiple testing because both tests need to demonstrate non-inferiority.

If non-inferiority has been established, superiority will be tested in the FAS based on the ITT population for the third primary endpoint. The hypothesis for the third primary endpoint is that the mean change in artifact size in MRI between 1-year follow-up and baseline in the magnesium-based group is different from the mean change in artifact size in MRI between 1-year follow-up and baseline in the titanium group. Superiority will be tested at the two-sided 5% test level. The Welch-type t-test will be used for judging superiority at the two-sided 5% test-level.

All secondary endpoints will be tested by appropriate tests and models exploratorily using the two-sided 5% significance level without adjustment for multiple testing.

Missing values for the primary endpoints will be imputed using MICE [46]. Missing data of scores from questionnaires will be handled according to the respective manual. As sensitivity analysis, a complete case analysis will be performed for the primary endpoints.

### Sample size calculations

Aim of the trial is to demonstrate non-inferiority of magnesium-based compression screws when compared with titanium Herbert screws for the fixation of scaphoid fractures. Standard deviations (SD) for the PRWE in the literature were all approximately 20 points [47]. Lange and Freitag [48] reported in their systematic review that many studies used a non-inferiority margin of 0.5 SD. This limit is used a non-inferiority margin for the planning of the scaphoid trial for the primary endpoint PRWE. This leads to the following assumptions for the sample size calculations for the first primary endpoint: Allocation ratio 1:1, type I error level 0.025 one-sided, power 0.9, expected difference between titanium and magnesium-based screws: Δ_1_ = 0, common standard deviation: σ = 20, non-inferiority margin: Δ_0_ = 10, drop-out 10%. To demonstrate non-inferiority under these assumptions, 94 patients per group, i.e., 188 patients in total are required for this trial.

If non-inferiority has been established for the first primary endpoint, the power to establish non-inferiority for the second primary endpoint, the safety endpoint is 90.64% under the following assumptions: type I error level 0.025 one-sided, event rate for magnesium-based and titanium screws: 0.9, non-inferiority margin: Δ_0_ = 0.15, drop-out 10%.

If non-inferiority has been established for the first primary endpoint and the second primary endpoint, the power to establish superiority for the third primary endpoint, the change in artifacts endpoint is virtually 100% under the following assumptions: type I error level 0.025 one-sided, expected difference between titanium and magnesium-based screws: Δ_1_ = 3.0, common standard deviation: *σ* = 1.0, sample size per group: *n*_*A*_ = *n*_*B*_ = 10, drop-out 10%, effective sample size per group: *n*_*A*_ = *n*_*B*_ = 9. The power is 56.41% if the mean difference is Δ_1_ = 1.0 instead of Δ_1_ = 3.0, and it is approximately 80% for Δ_1_ = 1.32.

The expected difference between the titanium and the magnesium-based group are substantiated as follows. Mean ± standard deviation artifact size were 8.4 ± 0.7 mm in the magnesium group and 12.9 ± 1.0 mm in the titanium group in the work of Sonnow et al. [15]. We assume a random reduction in artifact size in the titanium group of 1.0 mm and a halving of the artifact size in the magnesium group so that the difference is 3.4 mm, which is rounded to 3 mm. Due to the lack of additional data, we assume a common standard deviation of 1 mm, which is the standard deviation in the titanium group.

### Data management and data monitoring

Database and eCRF are developed, maintained and hosted by AMEDON GmbH. AMEDON GmbH will also perform data management. The trial database has been developed and validated before data entry based on standard operating procedures (SOPs). All changes made to the data are documented in an audit trail. The trial software has a user and role concept that can be adjusted on a trial-specific basis. The database is integrated into a general information technology infrastructure and safety concept with a firewall and backup system. The data are backed up daily. After completion and cleaning of data, the database is locked, and the data are exported for statistical analysis.

The data will be entered via the internet at the trial sites. Plausibility checks are run during data entry, thereby detecting many discrepancies immediately. Data management and monitoring will conduct further checks for data completeness and plausibility of data, and they will clarify any questions with the trial sites. These queries must be answered by the trial site without unreasonable delay. Further details are specified in the Data Validation Plan and in the Monitoring Manual.

Data management will be performed in compliance with AMEDON GmbH and in accordance to EN 14155 [49].

All relevant study data will be stored by the sponsor of the study. In the individual participating centers, the investigator files, study-related correspondence, patient identification list, consent forms and patient files will be retained for at least 10 years after the end of the study. Other in-house regulations or legislations demanding longer retention periods (e.g. radiation control regulation, radiation protection law) will be respected.

AMEDON GmbH is responsible for clinical onsite monitoring according to EN14155, written SOPs and the monitoring manual to ensure patient’s rights, patient’s security and reliability of trial results. For initiation, the trial site will be visited onsite by a clinical research associate. During the trial, sites will be visited at regular intervals depending on the rate of recruiting and data quality.

No audits are planned. However, to ensure correct execution of the study, audits may be conducted if necessary.

The trial is under medical direction. All staff members underlie medical confidentiality. Within a first training of staff and additional, consistently occurring staff trainings, the authorized clinic staff in the study sites will be instructed to handle all data as well as project-specific contents confidentially and to use all regular protection measures (recent version of antivirus-software, computer blocking by leaving the room, logout from eCRF after successful data transfer etc.).

### Governance

The whole project is supervised by a steering committee, which has regular meetings over the phone. An independent data monitoring committee (DMC) was not established because of the expected short accrual time. As the current study is conducted according to §23b of the German medical product act, no inspections of higher federal authorities are scheduled.

## Discussion

Scaphoid fractures are often fixated using titanium Herbert screws, which generally remain in the body. However, allergic reactions against titanium implants have been reported in few cases only [50, 51]. More important is screw removal which requires a second surgery. Biodegradable implants have the advantage that a second surgery for implant removal is not required. Polymer screws are biodegradable but their stability is substantially lower than the stability of titanium screws [8, 52, 53]. Magnesium-based screws combine the advantages of both materials, high stability together with biodegradability. MAGNEZIX® CS compression screws are the world’s first magnesium-based implants designed for use in biodegradable osteosyntheses applications in humans with market approval for the European Union and other countries [9]. An economic analysis for magnesium-based implants has been provided recently [21].

The non-inferiority of magnesium-based when compared with titanium compression screws has been demonstrated for hallux valgus surgery in a randomized controlled trial [20]. However, for scaphoid fractures only case reports have been published in which the use of magnesium-based implants is described [26, 27]. The randomized controlled SCAMAG trial will therefore generate high-level evidence in the use of magnesium-based compression screws for the treatment of scaphoid fractures.

## Trial status

The trial opened for accrual on Jan 2, 2018. First patient in was on Jan 4, 2018. Accrual is planned to be completed by the end of 2020.

## Additional files

Additional file 1: **Figure S1.** Study flow according to CONSORT statement. (PDF 1348 kb)

Additional file 2: SPIRIT 2013 and SPIRIT 2018 PRO Checklist applied to SCAMAG study protocol. (DOC 179 kb)

## Data Availability

Additional information on the study protocol will not be available because all relevant information is provided in this manuscript.

## Abbreviations

ADE: Adverse Device Effect
AE: Adverse Event
CONSORT: Consolidated Standards of Reporting Trials
CT: Computed Tomography
DASH: Disabilities of the Arm, Shoulder and Hand
DASH: Disability of the Arm, Shoulder and Hand
eCRF: Electronic Case Report Form
EN: European Norm
EQ-5D-5 L: Quality of life questionnaire
FAS: Full Analysis Set
FOV: Field Of View
FS: Fat saturated
GCP: Good Clinical Practice
ICH: International Conference on Harmonisation
ITT: Intention To Treat
KWS: Krimmer Wrist Score
MRI: Magnetic Resonance Imaging
PDw: Proton Density weighted
PP: Per Protocol
PRWE: Patient-Rated Wrist Evaluation
SADE: Serious Adverse Device Effect
SAE: Serious Adverse Event
SAP: Statistical Analysis Plan
SD: Standard Deviation
SOP: Standard Operating Procedure
SPIRIT: Standard Protocol Items: Recommendations for Interventional Trials
TSE: Turbo spin echo
WARP: Summary of methods to minimize the impact of metal implants on image quality
