# Supplementary figures and images for: Comparison of SCAphoid fracture osteosynthesis by MAGnesium-based headless Herbert screws with titanium Herbert screws: protocol for the randomized controlled SCAMAG clinical trial
Source: BMC Musculoskelet Disord. 2019 Aug 7;20:357. doi: 10.1186/s12891-019-2723-9 (PMC6685162; doi:10.1186/s12891-019-2723-9)

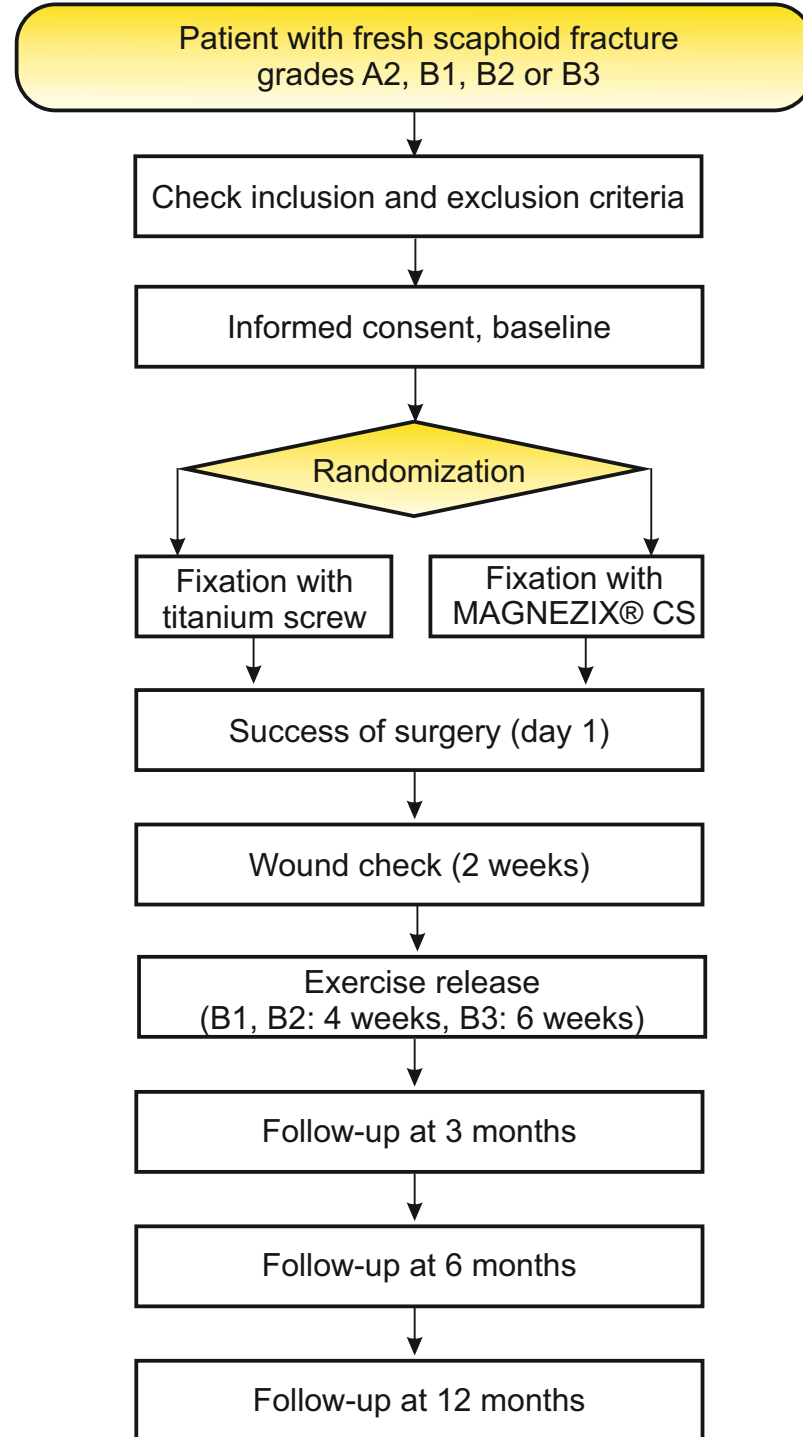

Supplement: Supplementary file 1 — Figure S1. Study flow according to CONSORT statement. (PDF 1348 kb) [file 12891_2019_2723_MOESM1_ESM.pdf]
