# Supplementary material for: Comparison of SCAphoid fracture osteosynthesis by MAGnesium-based headless Herbert screws with titanium Herbert screws: protocol for the randomized controlled SCAMAG clinical trial
Source: BMC Musculoskelet Disord. 2019 Aug 7;20:357. doi: 10.1186/s12891-019-2723-9 (PMC6685162; doi:10.1186/s12891-019-2723-9)
Supplement: Supplementary file 2 — SPIRIT 2013 and SPIRIT 2018 PRO Checklist applied to SCAMAG study protocol. (DOC 179 kb) [file 12891_2019_2723_MOESM2_ESM.doc]

SPIRIT 2013 and SPIRIT 2018 PRO Checklist applied to SCAMAG study protocol

| Section/item | Item No | Description | Explanation | Addressed in study protocol, section |
| --- | --- | --- | --- | --- |
| **Administrative information** | | |  |  |
| Title | 1 | Descriptive title identifying the study design, population, interventions, and, if applicable, trial acronym | Comparison of SCAphoid fracture osteosynthesis by MAGnesium-based headless Herbert screws with titanium Herbert screws: protocol for the randomized controlled SCAMAG clinical trial | Title page |
| Trial registration | 2a | Trial identifier and registry name. If not yet registered, name of intended registry | DRKS00013368, DRKS, Dec 04, 2017 | Title page |
|  | 2b | All items from the World Health Organization Trial Registration Data Set | See table at the bottom of this document | Provided as separate table |
| Protocol version | 3 | Date and version identifier | November 23, 2017, version 2.0 | Title page |
| Funding | 4 | Sources and types of financial, material, and other support | This trial is fully financed by Syntellix AG, Hanover. | Section 6 |
| Roles and responsibilities | 5a | Names, affiliations, and roles of protocol contributors | Journal article: MHK, SK, CP and AZ conceived the study. They initiated the study design, and KK and BB helped with the implementation. SK and CvF designed the image analysis protocol of the trial. AZ provided statistical expertise in clinical trial design and is responsible for the PRO content. All authors contributed to the refinement of the study protocol, reviewed and approved the final manuscript.  Study protocol: Prof. Dr. med. Martin H. Kirschner, Dr. med. Sören Könneker, Claudia Pieh and Prof. Dr. rer. nat. Andreas Ziegler conceived the study. They initiated the study design, and Dr. sc. hum. Katja Krockenberger and Bernard Brandewiede helped with the implementation. AZ provided statistical expertise in clinical trial design. All authors contributed to refinement of the study protocol and approved the final study protocol. | Section 3 |
|  | 5b | Name and contact information for the trial sponsor | Syntellix AG  Aegidientorplatz 2a  30159 Hanover  Germany  Represented by:  Prof. Dr. Utz Claassen (CEO)  Prof. Dr. med. Martin H. Kirschner (CTO)  Phone: +49 511 270 41371  Fax: +49 511 270 41379  e-mail: kirschner@syntellix.com | Section 2 |
|  | 5c | Role of study sponsor and funders, if any, in study design; collection, management, analysis, and interpretation of data; writing of the report; and the decision to submit the report for publication, including whether they will have ultimate authority over any of these activities | Sponsor is funder and was involved in the trial design. Trial management is shared between sponsor and AMEDON GmbH. The analyses will be performed by StatSol, and data interpretation by all parties involved. Report and publication writing will be with MHH and StatSol. The following text blocks are part of the study protocol: About publication: To meet the requirements of the Declaration of Helsinki, study results will be published irrespective of the outcome of the study. The scientific results will be published in international, peer-reviewed journals of the highest possible quality, irrespective of the study outcome. In addition, results will be presented at medical congresses and symposia. Publications will follow the CONSORT statement (Schulz et al., 2010) and its extension to abstracts (Hopewell et al., 2008).  For methodological reasons, results of the trial will be published only after study database closure. All reports and publication related to the study need to be coordinated with the trial biostatistician to avoid misinterpretation of statistical results. Conclusions need to be statistically secured and require approval of the trial statistician. | Section 3 |
|  | 5d | Composition, roles, and responsibilities of the coordinating centre, steering committee, endpoint adjudication committee, data management team, and other individuals or groups overseeing the trial, if applicable (see Item 21a for data monitoring committee) | Trial management, data management, central data management, regulatory affairs, monitoring: Bernard Brandewiede, AMEDON GmbH, Willy-Brandt-Allee 31c, 23554 Lübeck, Germany  Dr. sc. hum. Katja Krockenberger, AMEDON GmbH, Willy-Brandt-Allee 31c, 23554 Lübeck, Germany  Recruitment is expected to be completed 24 months after first patient in. Safety can only be fully evaluated 1 year after inclusion of a patient. Given the short recruitment time and the long safety evaluation period we refrain from establishing an independent Data Monitoring Committee (DMC) in this trial.  Pre-surgery images and images at 6 month follow-up will undergo review by two independent reference radiologists.  Evaluation of AEs and SAEs will be done independently by two experienced surgeons to judge causality (AE vs. ADE, SAE vs. SADE) | Section 2, Section 16.1      Section 18.2  Section 2, Section 18.1  Section 18.1 |
| Introduction |  |  |  |  |
| Background and rationale | 6a | Description of research question and justification for undertaking the trial, including summary of relevant studies (published and unpublished) examining benefits and harms for each intervention | See manuscript, background | Section 7 |
|  | 6b | Explanation for choice of comparators | See manuscript, background, study objectives | Section 7 |
| Objectives | 7 | Specific objectives or hypotheses | See manuscript, background, study objectives | Section 8 |
| Trial design | 8 | Description of trial design including type of trial (eg, parallel group, crossover, factorial, single group), allocation ratio, and framework (eg, superiority, equivalence, noninferiority, exploratory) | The SCAMAG trial is designed as a randomized, controlled, blinded observer, multicenter, post market clinical follow-up study for comparing MAGNEZIX® CS with titanium Herbert screws in patients in scaphoid fractures. Approximately 190 patients with scaphoid fractures will be randomized in a 1:1 ratio either to MAGNEZIX® CS or titanium screws.  In addition, a total of 20 patients treated in the centers near Hanover, the 10 first of each group will be included in the MRI part of the trial. | Section 9 |
| Methods: Participants, interventions, and outcomes | | |  |  |
| Study setting | 9 | Description of study settings (eg, community clinic, academic hospital) and list of countries where data will be collected. Reference to where list of study sites can be obtained | Patients will be recruited in 15 high-volume centers in Germany, each of which has experience in conducting clinical trials, treating patients with scaphoid fractures and the use of both MAGNEZIX® and titanium screws. All surgeons are trained in fixation of scaphoid fractures with titanium Herbert screws and MAGNEZIX® compression screws prior to the trial. | Section 10 |
| Eligibility criteria | 10 | Inclusion and exclusion criteria for participants. If applicable, eligibility criteria for study centres and individuals who will perform the interventions (eg, surgeons, psychotherapists) | Manuscript, Table 1 | Sections 11.1 and 11.2 |
| Interventions | 11a | Interventions for each group with sufficient detail to allow replication, including how and when they will be administered | Inclusion criteria and exclusion criteria will be checked in patients with scaphoid fractures who visit a study center. Patients fulfilling all inclusion criteria, but no exclusion criterion will be included in the study after providing informed consent. Usual preoperative preparation will be performed. Patients will be randomized to one of the two treatment groups after anesthesia. Surgical procedures will be done as established in the study center with minimal access as possible: percutaneous, minimal invasive or open approach dependent to the fracture type. After surgery, the success of reposition and osteosynthesis will be controlled via plain radiographs. The further treatment and aftercare will be performed strictly as recommended in the AWMF S3 guideline for scaphoid fractures (Deutsche Gesellschaft für Unfallchirurgie, 2015). According to the fracture type splinting and training will be performed with x-ray control. Before exercise release and before stress release after the period of exercising, clinical endpoints and questionnaires will be collected. In case of unsafe fracture healing a CT-scan will be done but at the earliest 9 weeks after surgery. Ultimately, there is a statement of bone healing 3 months (± 1 week) after surgery for every patient. For this study, additional follow-up points are 6 (± 2 weeks) and 12 months (± 4 weeks) after surgery with clinical endpoints and questionnaires. | Section 13 |
|  | 11b | Criteria for discontinuing or modifying allocated interventions for a given trial participant (eg, drug dose change in response to harms, participant request, or improving/worsening disease) | There are no prespecified stopping rules, but participation of a patient in the study is voluntary, and the patient may refuse to participate or withdraw from the trial, at any time, without penalty or loss of benefits to which the patient is otherwise entitled. | Section 20.5 |
|  | 11c | Strategies to improve adherence to intervention protocols, and any procedures for monitoring adherence (eg, drug tablet return, laboratory tests) | Not applicable because of surgical procedure. |  |
|  | 11d | Relevant concomitant care and interventions that are permitted or prohibited during the trial | Concomitant care is standard care according to the AWMF S3 guideline for scaphoid fractures (Deutsche Gesellschaft für Unfallchirurgie, 2015).. | Section 13 |
| Outcomes | 12 | Primary, secondary, and other outcomes, including the specific measurement variable (eg, systolic blood pressure), analysis metric (eg, change from baseline, final value, time to event), method of aggregation (eg, median, proportion), and time point for each outcome. Explanation of the clinical relevance of chosen efficacy and harm outcomes is strongly recommended | The first primary outcome will be the German version (PRWE-G) of the patient-rated wrist evaluation (PRWE) total score measured 6 months after randomization (John et al., 2008).  The second primary outcome will be the safety outcome which will be a composite endpoint, consisting of evidence of bone union until 6 months, no adverse device effect (ADE) during surgery, no ADE during wound healing, no reoperation and no serious adverse device effect (SADE) within 1 year after randomization.  Artifacts will be measured for titanium screws and MAGNEZIX® CS as described by Sonnow et al. (2017) to evaluate changes of artifact appearance over time between both groups. In detail, all artifacts will be assessed in an axial plane or reconstruction of the screw. In MRI artifacts aligning the y-axis will be considered, defined as the vertical axis of the scanner. As artifact appearance is expected to be symmetrical, measurement will be performed by creating a straight line through the outer boundaries of the artifacts and the central screw axis. The degree of artifact is defined as the diameter of the signal loss induced by the screw in MRI. When artifacts with various lengths are produced, the longest will be measured. This process will be performed in a total of three different axial slices of the screw, and the average value will be obtained. For better orientation and comparability, the artificial cartilage lesion will serve as reference, thus the slices in a similar position will be chosen.  The change in artifacts can now be defined as follows. Difference between maximum length of an artifact between 1-year follow-up and baseline. The third primary outcome is defined quantitatively as the extent of change in artifacts. The specific imaging modality for the third primary endpoint will be defined as early as possible during the trial and before inclusion of the last patient of the MRI part of the trial.  No additional explanation provided for relevance of the three primary endpoints as all are immediately clinically relevant.  Secondary endpoints listed in manuscript, methods, secondary endpoints. | Section 12 |
| Participant timeline | 13 | Time schedule of enrolment, interventions (including any run-ins and washouts), assessments, and visits for participants. A schematic diagram is highly recommended (see Figure) | Supplementary Figure 1 displayed in a separate file | Section 13 (Figure 1) |
| Sample size | 14 | Estimated number of participants needed to achieve study objectives and how it was determined, including clinical and statistical assumptions supporting any sample size calculations | Manuscript, methods, sample size calculations | Section 17.3.2 |
| Recruitment | 15 | Strategies for achieving adequate participant enrolment to reach target sample size | Access to patients through high volume centers, motivation letters, motivation phone calls. | Section 10 |
| **Methods: Assignment of interventions (for controlled trials)** | | |  |  |
| Allocation: |  |  |  |  |
| Sequence generation | 16a | Method of generating the allocation sequence (eg, computer-generated random numbers), and list of any factors for stratification. To reduce predictability of a random sequence, details of any planned restriction (eg, blocking) should be provided in a separate document that is unavailable to those who enrol participants or assign interventions | Randomization will be executed in a 1:1 ratio to MAGNEZIX® CS or titanium using stratified permuted block randomization (PBR) with variable block length. As recommended in the ICH E9 guideline (CPMP, 1998), center will be used as stratification variable. Randomization lists for the PBR will be generated using the randomization software RITA (Pahlke et al., 2004). | Section 15 |
| Allocation concealment mechanism | 16b | Mechanism of implementing the allocation sequence (eg, central telephone; sequentially numbered, opaque, sealed envelopes), describing any steps to conceal the sequence until interventions are assigned | Concealment of allocation will be guaranteed through central randomization within the electronic case report form (eCRF) according to Standard Operating Procedures (SOP). | Section 15 |
| Implementation | 16c | Who will generate the allocation sequence, who will enrol participants, and who will assign participants to interventions | Generation of randomization list: biostatistician; transfer of randomization lists into trial database: data manager; participant enrolment: surgeon; assignment: electronically; result displayed to surgeon | Section 15 |
| Blinding (masking) | 17a | Who will be blinded after assignment to interventions (eg, trial participants, care providers, outcome assessors, data analysts), and how | Blinded outcome assessor. | Section 18.1 |
|  | 17b | If blinded, circumstances under which unblinding is permissible, and procedure for revealing a participant’s allocated intervention during the trial | Not applicable | Not applicable |
| **Methods: Data collection, management, and analysis** | | |  |  |
| Data collection methods | 18a | Plans for assessment and collection of outcome, baseline, and other trial data, including any related processes to promote data quality (eg, duplicate measurements, training of assessors) and a description of study instruments (eg, questionnaires, laboratory tests) along with their reliability and validity, if known. Reference to where data collection forms can be found, if not in the protocol | Manuscript, table 2 | Section 13 |
|  | 18b | Plans to promote participant retention and complete follow-up, including list of any outcome data to be collected for participants who discontinue or deviate from intervention protocols | It is intended to follow-up all patients at scheduled follow-ups.  No special procedures have been established to promote patient retention. However, follow-up rates are generally high after fractures treated by a surgical procedure. | Section 13 |
| Data management | 19 | Plans for data entry, coding, security, and storage, including any related processes to promote data quality (eg, double data entry; range checks for data values). Reference to where details of data management procedures can be found, if not in the protocol | eCRF database | Section 19 |
| Statistical methods | 20a | Statistical methods for analysing primary and secondary outcomes. Reference to where other details of the statistical analysis plan can be found, if not in the protocol | Statistical analysis plan will be finalized prior to randomization of the last patient.  Manuscript, methods, statistical analysis. | Section 17 |
|  | 20b | Methods for any additional analyses (eg, subgroup and adjusted analyses) | Manuscript, methods, statistical analysis | 17.7 |
|  | 20c | Definition of analysis population relating to protocol non-adherence (eg, as randomised analysis), and any statistical methods to handle missing data (eg, multiple imputation) | Manuscript, methods, statistical analysis | Section 17.1 |
| **Methods: Monitoring** | | |  |  |
| Data monitoring | 21a | Composition of data monitoring committee (DMC); summary of its role and reporting structure; statement of whether it is independent from the sponsor and competing interests; and reference to where further details about its charter can be found, if not in the protocol. Alternatively, an explanation of why a DMC is not needed | Recruitment is expected to be completed 18 months after first patient in. Safety can only be fully evaluated 1 year after inclusion of a patient. Given the short recruitment time and the long safety evaluation period we refrain from establishing an independent Data Monitoring Committee (DMC) in this trial. | Section 18.2 |
|  | 21b | Description of any interim analyses and stopping guidelines, including who will have access to these interim results and make the final decision to terminate the trial | Neither interim analyses nor adaptations planned. | Section 17 |
| Harms | 22 | Plans for collecting, assessing, reporting, and managing solicited and spontaneously reported adverse events and other unintended effects of trial interventions or trial conduct | Safety is one of the first two primary endpoints. | Sections 12.1.2, 12.3, 16, 17.3, 17.6 |
| Auditing | 23 | Frequency and procedures for auditing trial conduct, if any, and whether the process will be independent from investigators and the sponsor | No audits planned. | Section 19.5 |
| Ethics and dissemination | | |  |  |
| Research ethics approval | 24 | Plans for seeking research ethics committee/institutional review board (REC/IRB) approval | Approval obtained. Ethics approval was obtained from the ethics committee of the Hanover Medical School on September 27, 2017, and its registration number is 7614. | Section 20.1 |
| Protocol amendments | 25 | Plans for communicating important protocol modifications (eg, changes to eligibility criteria, outcomes, analyses) to relevant parties (eg, investigators, REC/IRBs, trial participants, trial registries, journals, regulators) | Amendments will be submitted to the ethics committee and noted in the trial registry. | Section 20.4 |
| Consent or assent | 26a | Who will obtain informed consent or assent from potential trial participants or authorised surrogates, and how (see Item 32) | Treating surgeon | Section 13 |
|  | 26b | Additional consent provisions for collection and use of participant data and biological specimens in ancillary studies, if applicable | Not applicable | Not applicable |
| Confidentiality | 27 | How personal information about potential and enrolled participants will be collected, shared, and maintained in order to protect confidentiality before, during, and after the trial | The trial is under medical direction. All staff members underlie medical confidentiality. All personal and patient data are treated as confidential and will be stored only pseudonymized.  Within a first training of staff and additional, consistently occurring staff trainings, the authorized clinic staff in the study sites will be instructed to handle all data as well as project-specific contents confidentially and to use all regular protection measures (recent version of antivirus-software, computer blocking by leaving the room, logout from eCRF after successful data transfer etc.). | Section 19.7 |
| Declaration of interests | 28 | Financial and other competing interests for principal investigators for the overall trial and each study site | CvF, SK and PMV are employees of Hanover Medical School, which receives a grant from Syntellix AG for conducting the trial. CP is an employee of Syntellix AG, and MHK is board member of Syntellix AG. KK is an employee of AMEDON GmbH, and BB is CEO of AMEDON GmbH. AMEDON GmbH provides trial services to Synteliix AG. AZ is a consultant to Syntellix AG. |  |
| Access to data | 29 | Statement of who will have access to the final trial dataset, and disclosure of contractual agreements that limit such access for investigators | Access to final dataset to trial biostatistician.  Local centers are entitled to use the recorded data for additional scientific exploitation under their own name, but not before the main results have been published. | Section 20.9 |
| Ancillary and post-trial care | 30 | Provisions, if any, for ancillary and post-trial care, and for compensation to those who suffer harm from trial participation | Not applicable | Not applicable |
| Dissemination policy | 31a | Plans for investigators and sponsor to communicate trial results to participants, healthcare professionals, the public, and other relevant groups (eg, via publication, reporting in results databases, or other data sharing arrangements), including any publication restrictions | The scientific results will be published in international, peer-reviewed journals of the highest possible quality, and they will follow publication statements. In addition, results will be presented at medical congresses and symposia. For methodological reasons, results of the trial will be published only after study database closure. | Section 20.9 |
|  | 31b | Authorship eligibility guidelines and any intended use of professional writers | Manuscript authorship will be selected according to the requirements of the New England Journal of Medicine (http://www.icmje.org/).  SK and AZ will write the first draft of the manuscript. | Section 20.9 |
|  | 31c | Plans, if any, for granting public access to the full protocol, participant-level dataset, and statistical code | Full protocol available upon request from the corresponding authors. Access to participant-level data and statistical analysis code may not be granted. | Not applicable |
| Appendices |  |  |  |  |
| Informed consent materials | 32 | Model consent form and other related documentation given to participants and authorised surrogates | Provided in the study protocol | Section 21 |
| Biological specimens | 33 | Plans for collection, laboratory evaluation, and storage of biological specimens for genetic or molecular analysis in the current trial and for future use in ancillary studies, if applicable | Not applicable | Not applicable |

Table. All items from the World Health Organization Trial Registration Data Set

| **Primary Registry and Trial Identifying Number** | drks.de, registration number: DRKS00013368 |
| --- | --- |
| **Date of Registration in Primary Registry** | Dec 04, 2017 |
| **Secondary Identifying Numbers** | Registration number at the ethics committee of the Hanover Medical School: 7614 |
| **Sources of Monetary or Material Support** | The trial is fully financed by Syntellix AG. |
| **Primary Sponsor** | Syntellix AG, Aegidientorplatz 2a, 30159 Hannover, Germany  Represented by Prof. Dr. Utz Claassen (CEO) and Prof. Dr. Martin H. Kirschner (CTO) |
| **Secondary Sponsor(s)** | - Coordinating Investigator: Dr. Sören Könneker, Department of Plastic, Aesthetic, Hand and Reconstructive Surgery, Hanover Medical School (MHH), Carl-Neuberg-Str. 1, 30625 Hanover, Germany - Head of Executing Department: Prof. Dr. med. Peter M. Vogt, Department of Plastic, Aesthetic, Hand and Reconstructive Surgery, MHH, Carl-Neuberg-Str. 1, 30625 Hanover, Germany - Biostatistician: Prof. Dr. rer. nat. Andreas Ziegler, StatSol, Moenring 2, 23560 Lübeck, Germany - Trial Management, Central Data Management, Regulatory Affairs, Monitoring, Data Management: Bernard Brandewiede and Dr. Katja Krockenberger, AMEDON GmbH, Willy-Brandt-Allee 31c, 23554 Lübeck, Germany - Trial management: Claudia Pieh, Syntellix AG, Aegidientorplatz 2a, 30159 Hanover, Germany |
| **Contact for Public Queries** | Dr. Sören Könneker, Department of Plastic, Aesthetic, Hand and Reconstructive Surgery, Hanover Medical School (MHH), Carl-Neuberg-Str. 1, 30625 Hannover, Germany |
| **Contact for Scientific Queries** | Dr. Sören Könneker, Department of Plastic, Aesthetic, Hand and Reconstructive Surgery, MHH, Carl-Neuberg-Str. 1, 30625 Hannover, Germany |
| **Public Title** | SCAMAG: Magnesiumschrauben bei Kahnbeinbrüchen |
| **Scientific Title** | Comparison of SCAphoid fracture osteosynthesis by MAGnesium-based headless Herbert screws with titanium Herbert screws: protocol for the randomized controlled SCAMAG clinical trial |
| **Countries of Recruitment** | Germany |
| **Health Conditions** | Scaphoid fracture and indication for osteosynthesis by a Herbert screw fixation; fractures of types A2, B1, B2 and B3 according to Krimmer et al. 2000 Unfallchirurg |
| **Interventions** | **Intervention:**  Fixation with titanium Herbert screw.  **Control intervention:**  Fixation with MAGNEZIX® compression screw.  Treatment will follow German AWMF S3 guideline for scaphoid fractures. |
| **Key Inclusion and Exclusion Criteria** | **Inclusion criteria:**   - Indication for screw fixation of scaphoid fracture which is not older than 12 weeks (type A2, B1, B2, B3 by Herbert`s classification, modified by Krimmer). - Normal wrist function prior to fracture. - Age ≥18 years. - Written informed consent for trial participation and surgery.   **Exclusion criteria:**   - Previous surgery of the wrist, associated injuries, osteoarthritis, or Complex Regional Pain Syndrome (CRPS). - Simultaneous fractures of the forearm of both sides and those who will influence the postoperative care. - Known ligamentary concomitant injuries of the wrist on both sides and those who will influence the post-operative care. - Radiological findings of medium to high grade osteoporosis. - Intended or conducted spongiosa transplantation or bone graft transplantation during surgery - Pregnancy, suspected pregnancy or breastfeeding period. - Allergies to components of osteosynthesis material. - Participation in other clinical trials. - Central neurological deficits which do not permit a compliance to the trial, especially during follow-up. - For patients undergoing MRI: claustrophobia and metallic implants being contraindicative for MRIs.Participation in other clinical trials up to 30 days before inclusion in this trial. |
| **Study Type** | - Interventional - Randomized - Blinded observer - Parallel group - Multicenter - Randomization: permuted block randomization with variable block length and stratification by center; randomization electronically after complete patient registration using validated software; randomization result will only be transferred after complete electronic patient registration |
| **Date of First Enrollment** | January 04, 2018 |
| **Target Sample Size** | Approximately 190 in total, 95 per group. |
| **Recruitment Status** | Ongoing |
| **Primary Outcomes** | - First primary endpoint: The first primary outcome will be the German version (PRWE-G) of the patient-rated wrist evaluation (PRWE) total score measured 6 months after randomization (John et al., 2008).. - Second primary endpoint: The second primary outcome will be the safety outcome which will be a composite endpoint, consisting of evidence of bone union until 6 months, no adverse device effect (ADE) during surgery, no ADE during wound healing, no reoperation and no serious adverse device effect (SADE) within 1 year after randomization. - Third primary endpoint: The third primary outcome is defined quantitatively as the extent of change in artifacts between baseline and 1 year after randomization. Artifacts will be measured as described by Sonnow et al. (2017). |
| **Key Secondary Outcomes** | **Efficacy:**   - PRWE total score at 3 and 12 months. - PRWE domains pain and function at months 3, 6 and 12. - Disabilities of the Arms, Shoulder and Hands (DASH) total score (disability/symptoms) at 3, 6 and 12 months. - DASH module scores sport/music at 3, 6 and 12 months. - DASH module scores work at 3, 6 and 12 months. - Krimmer Wrist Score (KWS) total score at months 3, 6 and 12. - Domains of the KWS at months 3, 6 and 12. - Classified KWS (excellent, good, fair, poor) at months 3, 6 and 12. - Range of motion (wrist flexion, wrist extension, wrist radial deviation, wrist ulnar deviation, forearm supination) as measured by a goniometer at months 3, 6 and 12. - Grip strength as measured by a dynamometer at months 3, 6 and 12. - Bone union until month 6. - Quality of life (EQ-5D-5L) at baseline, 3, 6 and 12 months. - Time to return to work. - Time to return to recreational activities.   **Safety:**   - Adverse events, adverse device effects, serious adverse events, serious adverse device effects during surgery and wound healing. - Serious adverse events and serious adverse device effects during 1 year follow-up period. - Edema in MRI. |
